# Performance Evaluation of Rapid Test for *Schistosoma Mansoni* among School Aged Children in Mwanga District Council, Kilimanjaro Tanzania

**DOI:** 10.24248/eahrj.v8i1.746

**Published:** 2024-03-28

**Authors:** Seif Abdul, Victoria Masue, Magreth A. Mlemba, Rafaeli Massawe, Victor Mosha, Beatrice J. Leyaro, Sia E. Msuya

**Affiliations:** a Institute of Public Health, Department of Community Health, Kilimanjaro Christian Medical University College, Moshi, Tanzania; b Kilimanjaro Christian Research Institute, Moshi, Tanzania; c Institute of Public Health, Department of Epidemiology & Biostatistics, Kilimanjaro Christian Medical University College, Moshi, Tanzania; d Department of Community Medicine, Kilimanjaro Christian Medical Centre, Moshi, Tanzania

## Abstract

**Background:**

Schistosomiasis is an acute and chronic tropical disease caused by trematodes of the genus Schistosoma. It is a disease of public health concern and mostly affects developing countries of the tropics. According to WHO burden of the disease is as high as 80-85%, principally in sub-Saharan Africa. Although the majority of the infection is often linked with morbidity, it also results in considerable death. The overall annual mortality rate might exceed 200,000 people in Africa due to different complications of urinary and intestinal Schistosomiasis. Children are at a greater risk of acquiring the infection as well as reinfection, and this might cause growth retardation, anemia and low school performance.

**Objective:**

The study aimed at determining the prevalence of *Schistosoma mansoni,* associated factors and evaluating the performance of Point of Care Circulating Cathodic Antigen comparison (POC-CCA) against a routine method (formal Ether) of detection methods among school aged children at Mwanga District Council, Kilimanjaro Tanzania.

**Methodology:**

This was a cross sectional study conducted from April - June 2019 in Mwanga District Council. A minimum of 288 primary school children in Mwanga District were enrolled. Random sampling technique was used to select the participants. Interviews were conducted with study participants followed by single stool and urine sample collection. formal-ether concentration technique, urine dipstick and Point of Care Circulating Cathodic Antigen (POC-CCA) were used for stool and urine analysis. Data were entered and cleaned by using SPSS Version 20. Descriptive statistics were summarised using frequency and proportion for categorical variables and mean and standard dispersion for continuous variables. Logistic regression was used to identify independent factors associated with schistosomiasis. Any association with *P value* <.05 was considered significant.

**Results:**

A total of 288 participants were enrolled. The mean age of participants was 9.8 (±2.4) years. The prevalence of *Schistosoma mansoni* among the 288 students was 7.3% by formal ether method and 80.4% by POC-CCA. Social demographic characteristics, and hygiene practice assessed were not associated with *Schistosoma mansoni* in this study. Water source was statistically significantly associated with the prevalence of *Schistosoma mansoni*.

**Conclusion:**

The prevalence of *Schistosoma mansoni* among school aged children is low by using formal-ether concentration technique (routine method). The annual projects of deworming might have helped decrease the endemicity of the infection. This is due to regular deworming project as recommended by WHO. Despite various efforts which are done to deworm, school aged children are still at risk of acquiring infection, due to poor hygienic practice especially from water sources.

## BACKGROUND

Schistosomiasis, commonly known as bilharzia, is an acute and chronic tropical disease caused by trematodes of the genus Schistosoma.^1^ The schistosoma parasite is transmitted through a snail intermediate host with the human being the definitive host.^2^ There are 5 species of Schistosoma that infect humans, 3 species are common and unevenly-distributed worldwide which are *S. haematobium, S. mansoni and S. japonicum* and followed by *S. mekongi and S. intercalatum*.^3^

World Health Organization (WHO) estimates that globally about 207 million people are infected by *Schistosoma mansoni* and 700 million people are at risk of schistosomiasis infection in a year whereby 90% of cases occurring in the Sub-Saharan Africa.^4,5^ Due to this infection may result into 130,000 deaths every year in Sub-Saharan Africa.^6^

People infected with *Schistosoma mansoni* end up having bloody diarrhea, hepatomegaly associated with periportal liver fibrosis and portal hypertension. Other complications of esophageal varices and hematemesis.^6^ Among primary school children, a chronic infection with these parasites may results into chronic malnutrition and anemia leading to stunted growth and impaired learning ability.^7^

Factors associated with *Schistosoma mansoni* infection consist of poor socioeconomic status, change in climate and human water contact behavior.^8^ Children living in the developing countries are at higher risk since they live in areas with poor sanitation and most often spend time swimming or bathing in the water bodies contaminated with cercariae, the infective stages of Schistosomiasis. The hygiene and playing behavior in water bodies increases the risk of being infected by Schistosoma mansoni.^9^

WHO has recommended a periodic deworming program (using antihelminth drug) to all at-risk people in endemic areas without any previous diagnosis from the individual where by treatment should be given at least once per year when the baseline prevalence of Schistosomiasis in the community is between 20% and 50%, and given twice a year when the prevalence of Schistosomiasis in that community is over 50%. This should go together with snail host control, health education, hygiene promotion, access to safe water and sanitation improvement.^5^ Deworming training and program among school children in mainland Tanzania started in 2005.^10^ About 4 rounds of deworming have been done in Tanzania 7-14 years since 2005. The last cycle of deworming in Mwanga DC Kilimanjaro region among primary school children was done in 2018. Reliable data on burden of Schistosoma infection post mass school deworming program is scarce. This reliable data and associated risk factors are important to inform the performance of program action or intervention is still needed. On the other hand, the routine method for detection of *Schistosoma mansoni* remains to be stool analysis by formal-ether concentration technique, however Point of Care circulating Cathodic Antigen (POC-CCA) is recommended as the rapid method of detecting presence of *Schistosoma mansoni* in urine. This study aimed at evaluating the POC-CCA method that can be used to detect *Schistosoma mansoni*. Therefore, apart from the study aimed at determining the prevalence of *Schistosoma mansoni*, associated factor and comparison of detection methods among school aged children, it also intended to evaluate the performance of the POC-CCA as the rapid test of detecting *Schistosoma mansoni* compared Formal-ether concentration technique.

## MATERIALS AND METHODS

### Study Setting

The study was conducted in Mwanga DC primary schools in Kilimanjaro region. The district is one among 7 districts in Kilimanjaro region. The district is bordered by Kenya in Northeast, Moshi Rural District in Northwest, Manyara Region in Southwest and Same District in the South. According to the Tanzania National Census, the population of Mwanga District was 131,442 in 2012 (Census report, 2013). Mwanga District Council has 16 wards. The district has a total of 115 primary schools, where six (06) of them are private owned and 109 are owned by the government. The primary schools have a total number 26,594 students.

Of the 16 wards, two (02) wards, Kileo and Lang'ata were purposely selected as the study area. This was based on District Reports that they have irrigation scheme, river and big water bodies and had higher occurrence of reported signs and symptoms of Schistosomiasis.

### Design, Sample and Sampling

This was a cross sectional study conducted from April - June 2019 in Mwanga DC. The study population was primary school children from 5 randomly selected primary schools at Kileo and Lang'ata wards in Mwanga DC. Sample size was estimated by using the following formula; N = Z^2^P(1-P)/d^2^ and the total of 288 sample were computed using a prevalence of 64.3% from a study done in western Tanzania.

There are a total of 13 primary schools in the 2 selected wards; 10 in Kileo ward and 3 in Lang'ata ward. Simple random sampling was used to select the schools in the 2 wards. Five out of the 13 schools randomly selected were Kagongo, Kileo, Mnoa, Kivulini and Mkombozi primary schools.

### Participants Selection and Consent

In these five schools, 288 children (154 males and 134 were females) were voluntarily enrolled. Primary school children from standard one to standard seven whose parents/guardians signed a written informed consent and children who assented to participate were included in this study.

The procedures used to obtain sample units from each class; for those meeting inclusion criteria, for group (boys) at each class, numbers were written pieces of paper matching the total number of boys, folded and put them in the box. The box was shaken vigorously then boys were asked to pick pieces of paper, one at a time. Those that picked number 1 to 5 were invited to participate. The same procedure was applied to girls. The procedure was repeatedly used in all targeted classes and five schools.

Two days before the study at each school a meeting was held between research team, students and teachers, objectives were explained to teachers and students. Then each student from standard 1 to standard 7 was provided with a letter to take to their parents/guardians. Consenting parents/guardians were requested to sign the consent form giving permission for their children to participate in the study.

### Data Collection

#### Questionnaire

A pretested Swahili translated questionnaire was used. It had 5 main sections. Section I collected information on socio demographic, household income and asset ownership. Section II assessed availability of water at household and use, Section III asked for availability of toilet and uses, section IV was for hygiene practices and knowledge of schistosomiasis while section V was for symptoms and signs of Schistosomiasis. Questionnaires had both open and closed ended questions.

### Sample Collection

Single stool and urine samples were collected using dry, clean well-labelled plastic containers from each of the enrolled children. Approximately 5–10 ml of urine and 5–7 g of stool samples were collected. Subsequently, 10% formalin was added to the stool samples as preservative, whereas no preservative material was added to the urine samples. Urine samples were tested on the study area after collection by using POC-CCA rapid test for *Schistosoma mansoni* and urine dipstick for hematuria. Stool samples were stored in a cooler box for less than eight hours ready for transportation daily to the wet laboratory at Kilimanjaro Christian Medical University College (KCMUCo) for examination by using formal-ether concentration technique for *Schistosoma mansoni*.

### Sample Testing

Preserved stool samples were analyzed by using qualitative formal-ether concentration technique. Microscopy was used for examination of the Schistosoma eggs and standard data collection sheet for recording laboratory results.

Urine samples were also collected from the enrolled study children and were tested for *Schistosoma mansoni* Circulating Cathodic Antigens (CCA) using the CCA Urine Cassette assay. The preparation and examination of the urine samples were performed according to the manufacturer's instructions. The results of the test were read 20 min after adding the buffer. If the control bands did not develop, the result was regarded as invalid. In a CCA test band it can be read as positive or negative. In a urine dipstick the presence or absence of blood in the urine was read and the results were recorded into data collection sheet.

### Data Analysis

Data were entered and cleaned by using Statistical Package for Social Sciences (SPSS) Version 20. Descriptive statistics were used to summarize data; proportions for categorical variables and mean or median with respective measure of central tendency for numerical variables. Odds ratios with their respectively 95% Confidence Interval (CI) were used to measure the strength of association between Schistosomiasis and exposure variables (such as; socio-demographic, availability of water, putting on foot wear, defecation habits and bathing).

### Ethical Considerations

Ethical approval to carry out the study was obtained from Tumaini University Makumira, KCMUCo Research and Ethical Committee. We sought the permission to use primary schools and the letter and research proposal was presented to the District Education Officer and District Medical Officer of the Mwanga District council. After permission from the District Council and schools, Swahili translated informed consent form was used to obtain parents/guardians consent for the children to participate in the study and assent was sought from students.

Numbers were used in all data collection sheets to maintain confidentiality. A register linking participant number and a name was kept by principal investigator. Stool samples found positive were linked with student names at register and their diagnosis was written in referral form to nearby Health facility.

The information obtained was used for research purpose only and the study was not intended to harm any students and their names and results will be handled with confidentiality.

After collection and examination of stool samples results were returned to study participants within 7 days of sample collection and all study participants who were found with schistosomiasis were taken to a nearby health center for treatment.

Stool samples and other material used during processing were collected in biohazard bag and disposed by incineration.

## RESULTS

### Socio-demographic Characteristics

Participants' age ranged from 5-15 years, with mean age of 9.8 (±2.4) years. Among all participants 154(53.5%) were males, 208(73%) lived with both parents and only 23.7% were graded as rich (Table 1).

**TABLE 1:** Socio-Demographic Characteristics Among Participants (N=288)

Characteristics	n	%
Age in years (N=266)		
5–10	153	57.5
11–15	113	42.5
Mean ± SD	9.8 ± 2.4	
Sex		
Male	154	53.5
Female	134	46.5
Religion (N=287)		
Christian	161	56.1
Muslim	126	43.9
Class (N=287)		
1–3	141	49.1
4–5	97	33.8
6–7	49	17.1
Living with both parents (N=285)		
No	77	27.1
Yes	208	72.9
House hold size (N=282)		
≤5	134	47.5
>5	148	52.5
Number of children (N=282)		
≤3	156	55.3
>3	126	44.7
Social economic status (202)		
Poor	69	34.2
Middle	85	42.1
Rich	48	23.7

**TABLE 2:** Water Availability, Bathing Places and Swimming Activities (N=288)

Characteristics	n	%
Water source at home (N=278)		
Tap water	64	23.0
Stream/river/dam	214	77.0
Water proximity		
Near (≤ 30minutes)	236	81.9
Far (> 30min)	52	18.1
Reliability of water		
No	17	5.9
Yes	271	94.1
Bathing site (N=287)		
Home	184	64.1
River/dam	103	35.9
Swimming at river/dam (N=285)		
No	62	21.8
Yes	223	78.2
Fishing activities (N=286)		
No	204	71.3
Yes	82	28.7

### Water Availability, Bathing Places and Swimming Activities

Of the 288 students, only 64(23%) reported tap water as source of water at home and 103(35.9%) reported to bath at either river or dam. Moreover 221(76.7%) of the participants reported to swim in water bodies (river and dam).

### Toilet and Hygiene Practices

Almost all participants 285(99%) reported to have toilets at home, and 173(60.3%) of the toilets were pit latrines. Majority 261(90.6%) of the participants reported to have enough toilets at school. Availability of water for hand washing at home was 249(86.8%) and 258(89.6%) at schools, (Table 3).

**TABLE 3:** Toilet and Hygiene practices (N=288)

Characteristics	n	%
Toilet availability at home		
No	3	1.0
Yes	285	99.0
Type of toilet at home (N=287)		
Pit latrines toilets	173	60.3
Flush toilets	114	39.7
Toilet availability at school		
No	27	9.4
Yes	261	90.6
Family share toilet		
No	210	72.9
Yes	78	27.1
Urinate while swimming (N=231)		
No	189	81.8
Yes	42	18.2
Defecate while swimming (N=232)		
No	194	83.6
Yes	38	16.4
Availability of water for hand washing at home (N=287)		
No	38	13.2
Yes	249	86.8
Availability of water for hand washing at school		
No	30	10.4
Yes	258	89.6

### Schistosomiasis Results by Different Diagnostic Methods

Three different methods were used for diagnosis of Schistosomiasis. Only 21(7.3%) were positive in formal ether (a routine used method in the study area) and 91(33.1%) were positive in Urine dipstick while 150(54.6%) and 71(25.8%) were positive and trace, respectively in POCCCA method (Table 4).

**TABLE 4:** Schistosomiasis Results by Different Diagnosis (N=288)

Diagnostic method	n	%
Formal-ether method		
Negative	267	92.7
Positive	21	7.3
Urine dipstick (N=275)		
Negative	91	33.1
Positive	184	66.9
POC-CCA (N=275)		
Negative	54	19.6
Trace	71	25.8
Positive	150	54.6

### Concordance Between POC-CCA and Formal-ether Concentration Method

The overall percentage agreement between the two methods was 24.7% while the positive percentage agreement between two the tests was only 7.6% with formal ether missing many positive samples that POCCCA detected. The details are shown in Table 5.

**TABLE 5:** Concordance between POC-CCA and Formal-Ether Concentration Method

POC-CCA results	Formal Ether results		TOTAL
	Negative	Positive	
Negative	51	3	54
Positive & Trace	204	17	221
Total	255	20	275

### Prevalence of Schistosomiasis by different methods

Among 288 studied participants the prevalence of Schistosomiasis was 7.3% by formal ether method and 33.1% by urine dipstick. By POC-CCA the prevalence was 80.4% when trace results were considered as positive and 54.6% when considered as negative (Figure 1).

### Factors Associated with Schistosomiasis among Participants

In crude analysis among all studied factors only source of water and bathing sites were significantly associated with Schistosomiasis. After adjusting for other factors only source of water remained significantly associated with Schistosomiasis where by participants who reported to use water bodies (river/dam/spring) as the source of water at home had more than two times higher odds of having Schistosomiasis compared to those who use tap water. (OR:2.48; 95%CI:1.24, 4.96) (Table 6).

**TABLE 6:** Factors Associated with Schistosomiasis Among Participants (N=288)

	Prev-POC %	CRUDE OR (95%CI)	*p-value*	ADJUSTED OR (95%CI)	*p-value*
Age					
5–10	57.9	1		1	
11–15	49.1	0.70 (0.42, 1.15)	.163	0.64 (0.27, 1.52)	.313
Sex					
Male	54.1	1		1	
Female	55.1	1.04 (0.65, 1.68)	.860	1.01 (0.58, 1.77)	.962
Class					
1–3	60.7	1		1	
4–5	46.2	0.55 (0.32, 0.95)	.031	0.66 (0.30, 1.49)	.320
6–7	54.2	0.76 (0.39, 1.48)	.427	1.35 (0.44, 4.18)	.594
Water source at home					
Tap water	38.3	1		1	
Stream/river/dam	60.5	2.46 (1.36, 4.45)	.003	2.48 (1.24, 4.96)	.01
Bathing site					
Home	49.4	1		1	
River/dam	63.7	1.80 (1.08, 2.97)	.022	1.68 (0.90, 3.13)	.105
Swimming at river/dam					
No	50.9	1		1	
Yes	54.9	1.17 (0.65, 2.11)	.590	0.70 (0.33, 1.46)	.336
Dizziness					
No	56.5	1		1	
Yes	43.8	0.41 (0.16, 1.01)	.052	1.00 (0.42, 2.34)	.992

## DISCUSSION

The result of the study showed that the prevalence of *Schistosoma mansoni* by using formal-ether method and POC-CCA rapid method were 7.3% and 80.4%, respectively. Water source at home was statistically significantly associated with Schistosomiasis (*P*<.05).

The prevalence of Schistosomiasis in this study by using formal-ether concentration method (routine method) was 7.3% which is much lower compared to the prevalence of Schistosomiasis among primary school children reported in the study conducted in Ukara Island, which was 63.91% by using Kato Katz technique,^11^ this might be due to the difference in sensitivity of the methods with Kato Katz having higher sensitivity compared to the formal ether technique. Also the prevalence of Schistosomiasis by using POC-CCA method was 80.4% which is slightly lower compared to that reported in the study conducted in Mwanza region, Tanzania among primary school children which was 94.9% by using the same rapid method.^12^ However, this prevalence was much lower than results of the studies conducted in Jimma zone in Ethiopia (24.01%) and Kenya (76.8%) and Lake Victoria in Rorya Mara Region (84.01%) in Tanzania and this variation maybe due to long endemicity of the infection in communities, high dependence on the lake for different domestic and economic activities, low socioeconomic status and geographical conditions that favour survival of Schistosoma mansoni.^9,13–15^

In this study the methods used in determining the presence of *Schistosoma mansoni* infection were formal ether concertation technique and POC-CCA rapid method. The formal-ether concentration method (routine used method in the study area) is a qualitative test and is 85.0% sensitive compared to other methods such as Kato-Katz method and FLOTAC technique that are 77.4% and 67.7% sensitive, respectively in detecting *S.mansoni*.^16,17^ Although POC-CCA is sensitive compared to others in detecting eggs it is not preferable for egg intensity (quantification) as done in Kato-Katz technique which also shows the burden of the helminthes.^18^ The POC-CCA diagnostic technique with great sensitivity between 76.7% and 99.1%, and specificity of 74.2% might improve the detection of infections.^6^ The POCCCA is considerably more sensitive than other methods at low infection, also the ability of the POC-CCA rapid method to detect *Schistosoma mansoni* in mixed infections was unimpeded.^19^

Others researchers have shown significant association between male children and schistosomiasis, these findings are in line with our findings that shows males had 45% higher odds of getting the infection compared to females, and also the children from class 6 and 7 had higher odds compared to others. Also, a study done in Ethiopia shows similar results on males having higher odds which was statistically significant (*P*<.005) and also the aged above 15 years had higher odds compared to others.^20^ Children from households with river/dam as source of water had 2 times odds of having *Schistosoma mansoni* compared to those reported to use tap water. Improving availability of piped water and less contact with dam/irrigation water was associated with control or elimination of Schistosoma in China.^21^

## CONCLUSION

The prevalence of *Schistosoma mansoni* among school aged children is low by using formal-ether concentration technique (routine method) but high by using POC-CCA method. The annual projects of deworming might have helped decrease the endemicity of the infection. This is due to regular deworming program as recommended by WHO. Despite various efforts which are done to deworm school aged children, some are still at risk of acquiring infection especially from water sources due to inappropriate hygienic practice.

### Limitations

Despite children being among the most affected group the study faced challenges on collecting demographic information and other water usage habits.

### Recommendations

In order to reduce chances of missing Schistosomiasis patients' cases the government should consider the use of POC-CCA as routine method in endemic areas.

The deworming project in schools should be conducted at list annually.

Education on hygienic practices especially on water source and awareness on Schistosomiasis should be given to all school aged student.

We recommend that future studies should use more sensitive tests like POC-CCA.

Improve community–based education Schistosomiasis transmission and prevention as greater than a half of the students were unaware of Schistosomiasis.
